# Chemical Futures

**DOI:** 10.1007/978-3-030-57081-1_9

**Published:** 2020-10-14

**Authors:** Anita Hardon

**Affiliations:** University of Amsterdam, Amsterdam, Noord-Holland the Netherlands

## Abstract

Here we turn to the strategies that young people use to prevent chemical harms, not just those related to single chemicals but also those related to the feedback loops and compounding effects generated by the multiplicity of chemicals in daily life. *Chemical Futures* takes as an example youth activists in France, the Générations Cobayes, and their mobilization against endocrine-disrupting chemicals. We examine what contributes to the relative invisibility of toxic risk, pointing especially to the role of corporations in generating uncertainty about scientific evidence. The ChemicalYouth project engaged in a range of collaborative, youth-led projects that demonstrate the many ways youth may be engaged in “harm reduction from below.” We suggest that a ChemicalYouth 2.0 project might involve a wider range of researchers, advisors, and laboratories, to make more visible the multiple toxicities that make up young people’s everyday lives. Finally, we argue that governments should team up with youth and complement their efforts with “harm reduction from above” initiatives to regulate unsafe chemicals and support youths’ efforts to observe the effects of chemicals on their bodies and share information with others.

Think of what you did today. You woke up in the morning and got out of bed. First you put your left foot onto the carpet, the skin of the sole in direct contact with the molecules of the flame retardant that coats the fibers of your carpet, just like in every industrially manufactured carpet around the world. And then the right foot followed. You yawned and inhaled deeply the fine particles of plastic that combustion and friction had leached into the air. Perhaps they came from the scented candle you lit the night before, and the one before that? Or the countless plastic objects that had passed through your room in the past few days? Or maybe the minute compounds simply came in through the window with the warm breeze of the dense, bustling city where you live.

I am guessing, and this is a pretty good guess, that you brushed your teeth. You did so conscientiously, moving the brush in thorough tooth-wide strokes, making sure that the butylparaben, propylparaben, and triclosan—all preservatives put into toothpaste to make it last longer on supermarket shelves and in bathroom cabinets, and also well-known endocrine disruptors—penetrate well into your gums. Then you took a shower, washing with a gel, shampoo, and perhaps facial scrub if you were not in a rush. If you are a woman, you may have also used an intimate douche. Your rinsed most of the cocktail of parabens, phthalates, artificial scents, and colorants off your body, although some of these molecules, even if you could not feel or see it, had already penetrated your skin. You stepped out of the shower and dried your body with a towel that still contained some of the pesticides used to grow the cotton it is made of. After all, 16 percent of the worlds’ insecticides are sprayed on cotton crops. You then got dressed and applied some kind of deodorant, and for a second you might have worried, like a growing number of people around the world does, about the suggested links between cancer and the aluminum salts contained in most antiperspirants. Finally, you rubbed on some lotion that contains a highly polluting polyethylene glycol as emulsifier (no wonder it is so silky), and if on that particular day you wanted to make a good impression, you may have splashed a few drops of perfume or cologne on your neck. And then you got dressed. (Rios Sandoval 2019, pp. 21–22)

This is the introduction to Mariana Rios Sandoval’s PhD thesis “There Is Politics in Your Shampoo,” in which she describes young people who belong to the activist organization Générations Cobayes (Guinea pig generations). The Cobayes launched an “Éco-Orgasme” campaign to call on youth to avoid using sex-related products that contain potentially toxic chemicals, such as lubricants, and sex toys, as well as other products young people might encounter in dating, such as bed linens, processed foods, cosmetics, and lotions (Fig. 9.1).

**Fig. 9.1:**
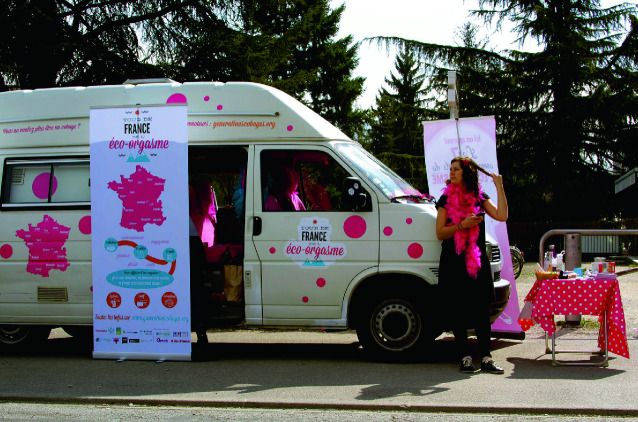
The Éco-Orgasme tour, a youth-led campaign in France (*Source* Photo taken by Mariana Rios Sandoval, March 2017, France)

The Cobayes’ main concern is the potential of such chemicals to cause endocrine disruption, infertility, and cancer. Manufacturers have denied this toxicity, but regulators have been slowly and partially recognizing the risk (Bellanger et al. 2015; Colborn et al. 1997; Tessaro 2020). The Cobayes call on their peers to avoid endocrine disruption by making their own soaps with safe ingredients, using reusable menstrual cups rather than sanitary pads, and buying locally sourced foods. By calling for consumer action, the Cobayes are making micro-level toxicities—not immediately apparent when consuming chemicals—into a tangible and graspable problem, one that can and should be addressed (Rios Sandoval 2019).

In this chapter we turn to the strategies that young people use to prevent chemical harms, not just those related to single chemicals but also, more broadly, those that comprise “chemical regimes of living” (Murphy 2008). Most of the ethnographies presented in this book concern deliberative acts of inhaling, swallowing, and injecting chemicals, as well as applying them to hair, faces, and bodies. We show that young people engage in such practices to achieve their aspirations for wellness and productivity at work, and that many of our interlocutors were not aware of how these practices simultaneously involve multiple, entangled toxicities. Two questions thread their way through this book: why do these youth trust that chemicals will not bring them harm, and what contributes to the relative invisibility of toxic risks?

Throughout this book we have pointed to the agency of youth in making chemicals do things for them through their situated practices, highlighting the creativity of their do-it-yourself chemical regimes, and the dense webs of human relations and things in which chemical efficacies and ways of knowing take shape. In these constellations of doing and knowing, chemicals should be understood as fluid (Hardon and Sanabria 2017). We have described how, across the field sites, young people viewed their skin tone, body shape, gendered identity, and mental and creative potential as modifiable through situated chemical practices. They tinkered with dosages, shared experiences, and substituted products in order to achieve desired states of being, while also moderating their use in order to prevent adverse effects.^1^ Having referred throughout to their risk-mitigating practices as “ harm reduction from below,” in this chapter we propose that youth should be supported with “ harm reduction from above.”

## In Search of a Good Life

In the seven empirical chapters, we discussed three general reasons for using chemical products. The first pertains to wellness: the young people we spoke with used chemicals to be attractive, healthy, connected, happy, and muscular; the second concerns desires to achieve diverse gendered ways of being in the world; and the third entails enhancing work capacity, productivity, and creativity. We noted how the positive potential of chemicals for these projects is amplified through advertising, tailored face-to-face sales, and online sharing, and is enabled by the easy accessibility of products in malls, pharmacies, and corner stores.

Youth’s engagement with wellness and beauty products suggests a pervasive trust in and reliance on their beneficial effects (see also Rodrigues 2016). Chemicals gave our interlocutors a sense of control as they faced multiple insecurities in their everyday lives, especially the work-related precariousness that was present across our fieldwork sites although in different ways. The daily engagements of young people with multiple chemicals is fueled by a neoliberal global economy, in which working conditions are characterized by flexibility and the lack of worker protections, causing job insecurity (Beck 2000; Harvey 2005; Kallenberg 2009; Vallas and Prener 2012). In addition to seeking a good education, youth invest in their bodies and minds (Anagnost et al. 2013), and acquire skills to be able to compete in an increasingly precarious labor market (Gershon 2017). Advertising campaigns feed on young people’s insecurities about their ability to accomplish all this, depicting cosmetics, cigarettes, energy drinks, and supplements as the means through which they can gain confidence and succeed in life (Chua 2000).

Growing up in a post-industrial era, their lives have overflowed with chemicals promoted by a multi-billion dollar beauty, wellness, and supplements industry. The industrial practices involved are under-regulated in neoliberal political constellations that favor economic progress and protect corporate interests. All sorts of claims are permitted in advertisements, as long as they do not make medical promises. In the absence of information on the potential toxic effects of the chemicals contained in these products, young people observed their own bodies to see what chemicals did, and they shared their experiences with others. If products didn’t work or had unwanted effects, they tried out new products and other brands.

Social theorists argue that in times of “uncertain global conditions” (Misztal 1996, p. 6), people increasingly rely on trust, which Misztal defines as “believ[ing] that the results of somebody’s intended action will be appropriate from our point of view” (p. 24). The mediation of experiential knowledge by peers, sales people, and multilevel marketeers, we observed, plays a key role in building such trust. Indeed, the “concreteness” of face-to-face communication fosters it (Brown and Calnan 2012). Thus, we argue that the amplification of benefits of products through advertising, along with the interpersonal relations through which young people try out chemicals and share information on them, together shaped our interlocutors’ overall trust in the chemicals that pervaded their lives.

Trust is typically described in social science literature as a benign dynamic, involving productive reciprocity. Here, we find it has a darker side. While providing our interlocutors with tools to confront the uncertainty in their lives, mechanisms of trust also rendered toxic risks of chemical invisible. Our analysis suggests that our interlocutors’ trust in the products that they used was unwarranted and even harmful. Interpersonal relations can be manipulated (Skinner et al. 2014); in our field sites, this occurred when companies paid youth to market chemicals to their friends, peers, and family. Our youth ethnographers observed these dynamics in multiple contexts, for example, where youth are paid to use their social media accounts to promote e-cigarettes to their peers (Chapter 10.1007/978-3-030-57081-1_3), or are lured to sign up to sell supplements with exaggerated health claims in multilevel marketing schemes, capitalizing on their networks of friends and family to make a living (Chapter 10.1007/978-3-030-57081-1_7).

In many cases, our interlocutors ended up worse off than when they started using chemicals, which they believed would help them achieve their aims in life. Whitening exfoliants harmed some of the young women working in karaoke bars, and their disfigured skin made it harder for them to attract clients. Potent painkillers used by sex workers to feel confident ended up causing dependency on the drugs, forcing them to keep working to maintain their drug habit. E-cigarettes used by youth as safe alternatives to cigarettes cause severe pulmonary conditions and can even be lethal. The overconsumption of energy drinks left low-income workers exhausted, while using high dosages of hormones to modify their bodies put transgender people at risk of getting cancer later in life. Buying supplements for resale through multilevel marketing companies left hopeful entrepreneurs in debt. Though government authorities may warn about the addictive potencies and toxicities of cigarettes and narcotic substances, our interlocutors were kept in the dark on the risks of cosmetics, pharmaceuticals, supplements, and e-cigarettes, all of which—in part because they are accessed easily in groceries and online stores—were widely considered safe. While our ethnographies reveal that young people across our sites sought to mitigate immediate chemical harms, the effects of the multiplicity of toxic exposures on their future health tended to escape their attention, precisely the problem the Cobayes sought to address.

## Regimes of Invisibility

Social scientists are increasingly examining and theorizing how chemical toxicities make up our everyday lives (Murphy 2008; Fortun 2012; Davis and Abraham 2013; Fortun et al. 2014; Povinelli 2017; Roberts 2017) and how they are governed by what Fortun and colleagues (2014) call “ regimes of invisibility.” Immense resources are invested in keeping the toxicity of chemicals secret, to avoid state regulation and scientific scrutiny. Corporations prioritize their sales, while neoliberal governments protect commercial interests. Chemical manufacturers oppose precautionary measures, they question the methodologies used to measure toxicities, and exaggerate scientific uncertainties with the aim of sabotaging decision making (Markowitz and Rosner 2002; Homberg & Vaupel 2019). Fortun and colleagues (2014) note that, even in a world of big data and widespread surveillance, some things seem to systematically “fall off the radar,” such as the monitoring of air and water quality, and the health impact of toxicants. Such invisibility obscures the “synthetic toxic relations” that surround us (Murphy 2008).

Proctor (2011) coined the term “agnotology” to refer to the deliberate strategies of making things invisible. The term foregrounds how uncertainty is not simply the absence of knowledge; rather it may be actively produced by social practices (Sanabria 2016). Proctor (2011) illustrates how agnotology works in an incisive historical study that shows how Big Tobacco diffused evidence that smoking was associated with lung cancer. Tobacco companies funded researchers to produce divergent interpretations of that evidence, in order to create uncertainty and prevent regulatory action, while simultaneously engaging in public campaigns that represented smoking as freedom and an adult choice, which created a political smokescreen in which regulatory action became hard to justify. Chapter 10.1007/978-3-030-57081-1_3 shows how, facing a global treaty on tobacco control, companies shifted their communication strategy to the promotion of “ safer nicotine products,” a term that renders e-cigarette toxicities invisible.

Similar regimes of invisibility have been documented in public debates on the safety of cosmetics, such as Johnson’s Baby Powder as we discussed in Chapter 10.1007/978-3-030-57081-1_5. Lawyers, representing US women who suffered from cancers associated with the product, reviewed company communications and found that Johnson and Johnson hand known for more than 40 years that its talc may be contaminated by asbestos, a potent carcinogenic (Rabin and Hsu 2018). In hundreds of pages of internal company documents, executives worried about the consequences of admitting to regulatory authorities that their product was unsafe. The company defended its commercial interests by discrediting research on the presence of asbestos, while also proposing new ways to measure the purity of the product; evidence of these efforts was unearthed by the *New York Times*, after obtaining company documents through the US Freedom of Information Act.

In Chapter 10.1007/978-3-030-57081-1_7 we discussed how the supplements industry also produces regimes of invisibility, by successfully preventing more stringent oversight on safety and efficacy by the US FDA. Highly orchestrated lobbying efforts to prevent stronger regulation involved doctors, who argued patients had rights to use supplements and vitamins in complementary medical treatments, and consumers, who fought for their freedom to use these “natural” products (Offit 2013; Scrinis 2013; Cohen 2016). As such products are now able to acquire a status of “generally recognized as safe,” the market for them is growing rapidly worldwide, driven by aggressive promotion of their benefits through social media and multilevel marketing . The products are promoted as protective shields for metabolic conditions, such as diabetes and cardio-vascular conditions, the occurrence of which is exacerbated by fast food diets containing high levels of salt and sugar (Scrinis 2013). Marketing their products as prevention, the supplements industry helps render invisible the negative role that food corporations play in current epidemics of metabolic diseases, while reinforcing industry rhetoric that individual choices determine health (Moodie et al. 2013; Sanabria 2016).

The agnotological mechanisms that take place in Big Pharma-sponsored trails of psychiatric pharmaceuticals produce ignorance that makes it possible for psychiatric medications to be approved by regulatory committees despite their potential for severe adverse effects (Healy 2012; Medawar and Hardon 2004). For example, when clinical trials funded by Eli Lilly and Company showed an increased risk for suicide among children taking Prozac, the manufacturer decided to not report this risk to authorities. Healy (2012) states, “When a company is faced with defending of a brand that is essential to its survival, commercial logic dictates that it will take any steps necessary to preempt the emergence of a hazard, including doctoring the evidence” (p. 53). Similar suppression of evidence has occurred in other pharmaceutical domains. For example in the field of pain medication, Purdue Pharma has been charged of suppressing information on the addiction potential of slow-release oxycontin, thereby fueling the current opioid crises in the United States (Lexchin and Kohler 2011; Kanouse and Compton 2015). We saw in Chapter 10.1007/978-3-030-57081-1_2 how similar epidemics of addiction occurred in Indonesia, where our interlocutors bought potent pain killers over the counter to enable them to work without realizing that the pills can cause dependence.

These industry-sponsored regimes of invisibility make it near impossible for youth to fully understand the risks of using chemicals to achieve their aspirations for wellness, sexuality, productivity, and creativity. The Cobayes address this by drawing attention to the potential toxicities involved in everyday chemical regimes. They encourage youth to exchange their daily chemical products for safer alternatives, efforts that, along with other practices of youth to mitigate harm, we label “ harm reduction from below.”

## Harm Reduction from Below

Strategies and efforts to reduce harm from below can involve a multiplicity of collaborative strategies that recognize young people’s chemical regimes of living and mobilize embodied resistance to the conditions of precarity which perpetuate chemical harm (cf. Medina 2013; Sabsay 2016). Harm reduction from below, as we propose it, builds on young people’s existing interest in mitigating the risks of chemicals, the hybrid nature of the networks in which they experiment with chemicals, and the protective nature of the social networks in which young people experiment with drugs. It amplifies the cautionary tales shared by young people online, supports their modes of self-regulation, and counters the exaggeration of benefits by industry. It also seeks to prevent adverse effects by drawing attention to less tangible, slowly accumulating toxicities; we can understand the campaigns of the Cobayes in this light, playfully working against the “merchants of doubt” (Markowitz and Rosner 2002), to make such toxicities visible.

Harm reduction programs emerged in the 1980s to prevent the transmission of HIV in communities of injecting heroin users, and since have targeted a wide range of substances, including speed, cannabis, nicotine, and alcohol. Harm reduction programs offer safer means to administer substances, such as clean needles to inject heroin or patches to deliver nicotine, and they promote safer chemicals, such as using buprenorphine or methadone to replace heroin. But they are not usually designed from “below.” Harm reduction efforts have tended to locate chemical risk in the actions of individuals, calling on heroin users, ( cannabis) smokers, and binge drinkers to take responsibility for their own health (Hardon and Hymans 2016). Our research leads us to conclude that such efforts threaten to misfire, or be misunderstood by youth, because they fail to build on the ways that young people already exchange experiences and self-regulate drug use in face-to-face and online social collectives.

**Fig. 9.2:**
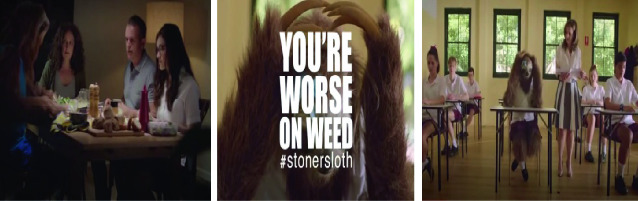
Screenshots form StonerSloth Campaign Videos (Aa4398743873 2015) (*Source* Photos taken by Anita Hardon, the Netherlands, April 2020)

For example, in New South Wales (Australia), a 2015 government-led harm reduction campaign had as its slogan: “You’re Worse on Weed.” The creative design firm Saatchi and Saatchi was hired, and they developed three YouTube videos featuring a human-size sloth named Jason (compilation uploaded by YouTube user “Aa4398743873” [2015]). The videos depict Jason’s siblings and peers responding to him when he is stoned in diverse circumstances (a dinner table, classroom, a party). In one, Jason sits at the dinner table; his mother asks him to pass the salt, and he reacts confusedly and passes the salad instead. His sister, apparently disgusted, calls him “Stoner Sloth.” The videos appear to be intended to make cannabis users fear being similarly shamed (Fig. 9.2).

The campaign failed (Nudd 2015), though, because youth thought the videos were hilarious and started sharing them through social media for laughs. In comments posted on the YouTube channel, where the videos are shared, a commenter named Milos asks, “is this video a joke or what?” and Richard answers, “I have absolutely no idea. I just find it unreasonably funny” (Aa4398743873 2015). The campaign seems strangely out of touch, not only with global moves toward legalizing cannabis as a relatively safe drug but also with young people’s capacity to moderate their use. Their capacity to do so is increased if they are adequately informed of risks, for example, regarding the high THC content of many varieties of cannabis, or of potential lung toxicity when vaping the substance (Chapter 10.1007/978-3-030-57081-1_3).

With their focus on individual behavior and on the risks of addiction and overdose, many such educational campaigns against alcohol, drugs, and smoking downplay the normalized status of the substances. They also fail to acknowledge the way such substances are embedded in and foster social relations, and that they can generate positive feelings such as relaxation, excitement, and pleasure (Malins 2004; Hunt et al. 2007; Duff 2008; Martinic and Measham 2008). Failing to recognize what many young people consider to be the benefits of substance use, and failing to build on young people’s collective self-regulation strategies, the effectiveness of harm reduction programs is reduced (Hardon and Hymans 2016).

In developing our own harm reduction from below efforts, we built on successful strategies undertaken in Amsterdam, where experimenting youth advised each other on harm reduction techniques, such as using micro-gram scales and “allergy-dosing” new kinds of drugs (Chapter 10.1007/978-3-030-57081-1_2). Through online forums, this community was able to adapt to challenges, for instance through substance warnings and providing advice for members experiencing problems (van Schipstal et al. 2016). We were impressed by the pragmatic way that municipal authorities supported young people in their efforts to reduce harm, including providing access to testing facilities where young people could have their ecstasy pills and LSD blots tested for purity, and training peer supporters to keep an eye on drug users during festivals. These pragmatic harm reduction efforts had been developed as an alternative to individual-oriented, nudging approaches to harm reduction, and to drug policies that stigmatize, criminalize, and marginalize drug users.

Concerned about the precariousness of drug users in Indonesia, where there is no effort to reduce harm from above or below, we organized deliberative dialogs between drug experts and youth. In one of these sessions, a pharmacy professor from the University Hasanuddin in Makassar met with a group of heavy drug users. He walked them through his understandings of the pharmaco-kinetics of the drugs that they used, pointing to the hard work that their livers needed to do to metabolize the large dosages of psychoactive prescription drugs that they used on a daily basis. He asked them whether they had friends who had died, and what the cause was. When he was told by the drug users that a friend had died of hepatitis, he asked follow up questions, eventually explaining that “becoming yellow,” could be caused by liver failure and discussed the stresses of heavy drug use on the kidneys of users. Our respondents had been evaluating the safety of drugs, and mitigating harm, by observing acute effects. They considered their drug-use practices safe enough if they woke up the next morning feeling reasonably well. But the slower, and much less visible effects on their livers and kidneys had not been part of their experiential knowledge. By taking their experiences seriously and creating space to discuss different perspectives on risk, we were able to create a reflective space that allowed many of our respondents reconsider their drug-use habits.

**Fig. 9.3:**
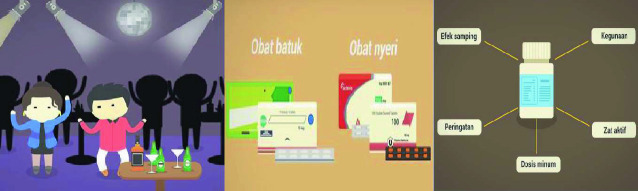
Three images from the Kok Bisa/ Pamflet YouTube video *Apakah Minum Obat Bisa Membahayakan Kesehatan Kita?* (Can taking medicines cause harm?)

To reach a larger audience, we developed a harm reduction from below campaign with two youth communication collectives in Jakarta, Pamflet and Kok Bisa. They proposed to spread “tales of caution” on social media, a strategy that had proven successful in other campaigns. This collaboration resulted in a short YouTube video on the growing use of psychopharmaceutical drugs and their adverse effects, conveying that (1) all pharmaceuticals can have side effects and should be used with caution, and (2) product packaging contains important information about pharmaceutical content, indications for use, dosage, and side effects. As of April 2020, this experimental 3-minute educational video had been viewed 360,000 times and attracted nearly 8000 likes (Fig. 9.3).

## Confronting Toxic Uncertainties

The harm reduction from below approach that we propose intends to address less visible chemical exposures and slow toxicities, as in the Cobayes’ campaigns. Murphy’s (2008) conceptualization of chemical regimes of living provides a useful framework for analyzing slow toxicities, related to the multiplicity of synthetic chemicals used by young people every day. The concept invites us to examine the totality of soaps, creams, supplements, e-cigarettes, hormones, and psychoactive substances that young people inhale, ingest, inject, and rub on their skin on a daily basis. It asks us as well to attend to the complex feedback loops through which chemicals pass through our bodies, combining with environmental pollutants—pesticides, factory emissions, sewage, and transportation exhaust—that then re-enter our bodies entangled with food, water, and air (Roberts 2017; Hardon and Sanabria 2017).

Attending to toxic entanglements is not easy, especially given the regimes of invisibility described above. Over the past two hundred years of industrialization, societies and scientists have struggled to keep track of the safety profiles of chemicals, both the long-used ones and the constant stream of new chemicals, used in agriculture and homes, and in cosmetics, supplements, and pharmaceuticals. More often than not, when chemicals appear on the market, their risk profiles are poorly understood and are used for many years before evidence on harm emerges (MacKendrick 2018; Homberg and Vaupel 2019).

Moreover, knowledge of toxicities is generated by researchers working in a range of disciplines, each of which producing field-specific knowledge with their own measurement techniques, resulting in their own “agential cut” (Barad 2007, p. 176) on the problem of the toxic entanglements. These disciplines include environmental sciences, ecology, epidemiology, reproductive medicine, dermatology, forensic and military medicine, pulmonology, and occupational health. Evidence of toxicities does not necessarily travel across disciplinary domains, as we see in the case of e-cigarettes. Chemicals used to flavor e-cigarettes had been identified as toxic by occupational health scientists who conducted studies of workers’ health in the food industry, but this toxicity was not associated with e-cigarettes until pulmonologists noted that vaping could cause severe lung pathology.

Measurements of chemical toxicities furthermore change over time. For example, in the early twentieth century, animal studies were used to define threshold levels below which substances were deemed safe. When I studied biology in the early 1980s, we were taught that such thresholds could be defined by measuring the LD_50_, the lethal dose at which half the population of test animals would die due to exposure to the substance. Research on the teratogenic and mutagenic effects of chemicals in the latter decades of the twentieth century, however, demonstrated that some chemicals could disrupt endocrine systems and cause cancers even at very low levels of exposure.

How can we make poorly understood slow toxicities visible? We are inspired by the Éco-Orgasme campaign of the Cobayes, designed to raise awareness of the multitude of chemicals in youth’s everyday lives that can cause endocrine disruption, the effects of which are not immediate and are hard to perceive. The campaign attracted youth by playfully registering their chemical sexualities. This is what Camille, a 24-year-old Cobayes activist, told an audience of French students about what they could expect:

Is there anyone here that already knows the eco-orgasm? You came here because you saw the word orgasm in the title of this morning’s workshop? Is that it? [Audience giggles.] Ah, yes, very good choice, very good choice indeed. Well, I will begin by introducing us briefly; here we have Thibault who is an eco-orgasm giver, Alice too, she gives eco-orgasms also. … What we will present this morning is how to go from a nice, simple orgasm, to an *orgasm de ouf* [crazy, cool], an *orgasm écolo*, inventive, creative and sustainable, and voilà, that’s the program for this morning until 12:30. Is that OK with you?”. (Rios Sandoval 2019, pp. 83–84)

**Fig. 9.4:**
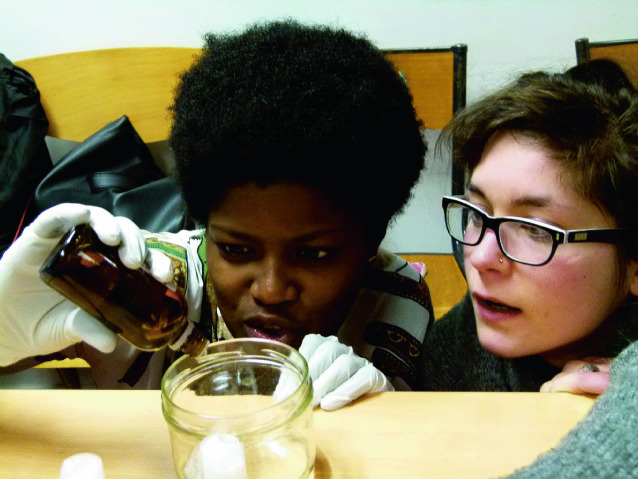
Do-it-yourself preparation of make-up remover (See more images from Rios Sandoval’s fieldwork at https://www.chemicalyouth.org/#/projects/there-is-politics-in-your-shampoo) (*Source* Photo taken by Mariana Rios Sandoval, March 2018, France [Rios Sandoval 2019])

Rios Sandoval describes how this highly interactive event, which traveled to many French university towns, warned youths about toxic chemicals hidden along the “*parcours de la drague*” (cruising route). The event was highly structured, with 15 minutes of sharing facts about the mechanisms and consequences of exposure to endocrine-disrupting chemicals, followed by theatrical sketches to engage the audience. The sketches played out practical solutions, such as a dazzling date with a meal free of pesticides, and condoms and self-made lubricants free of endocrine-disrupting chemicals. Rios Sandoval (2019) writes that her interlocutors “apprehend toxicity through the re-appropriation of production chains, a mutually triggered and reinforced process. Do-it-yourself practices are tools for young people to intervene in the toxic relations making up everyday toxicity” (p. 251) (Fig. 9.4).

We found video and photo projects to be extremely useful in making visible young people’s chemical regimes of living. The ChemicalYouth project, committed to the implementation of multimodal anthropology (Collins et al. 2017) has provided an experimental space for several visual arts projects. In Cagayan de Oro, for example, we asked students participating in the field school of the Anthropology Program of the University of the Philippines to take pictures using their mobile phones of chemical information, and access points in town. As the photos contained GPS coordinates, participants were able to make an interactive ChemicalYouth map of the city.^2^

**Fig. 9.5:**
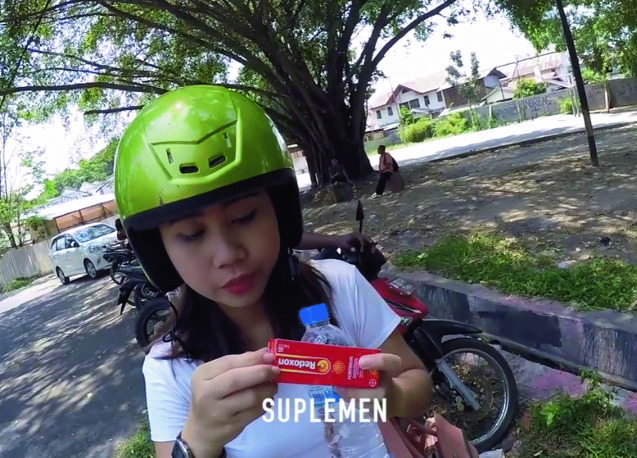
Screenshot from a GoPro exploration of the chemical lives of students (https://www.chemicalyouth.org/#/projects/a-day-in-the-chemical-lives-of-manadonese-youth) (*Source* Photo taken by Anita Hardon, April 2020, the Netherlands)

In Manado, the youth ethnographers took stock of all the chemicals consumed by students in one day using a GoPro camera, a video of which can be found on our website, www.chemicalyouth.org (Fig. 9.5).

We also worked with young filmmakers from to produce the documentary *Sweet Medicine* on the dark-side of multilevel marketing of supplements (see Chapter 10.1007/978-3-030-57081-1_6),^3^ and two mini-documentaries that examined skin-whitening practices in Manila, contrasted these with the embrace of brown skin in a surfing community in the south of the Philippines (see Chapter 10.1007/978-3-030-57081-1_5).^4^

We were also inspired by the potential to make visible chemical regimes of living that were presented in the evocative documentary *Toxic Beauty* (Ellis 2019). This documentary addresses the lack of regulation of cosmetics, and presents the litigation by a group of women claiming that Johnson’s Baby Powder caused the cancer they have suffered. The documentary maker presents Nguyen, a young Vietnamese woman pursuing a master’s degree in medical sciences at Boston University, who has become worried about the toxic effects of the multiplicity of cosmetic products, including whitening creams that make up her chemical regime of living. In her classes she learns about the endocrine-disrupting effects of parabens and phthalates found in the cosmetics that she uses. She also writes a term paper, inspired by Stein and Lourie’s (2019) *Slow Death by Rubber Duck*, about experiments to lower the level of toxins in one’s everyday life. Nguyen self-experiments to lower the amount of parabens and phthalates in her body, listing all the contents of the 27 different products that she usually uses, and measuring chemicals in her urine with a “ chemical body burden kit” distributed by the Silent Spring Institute. The test involves collecting a urine sample on three consecutive days: on the first day, she applies her regular skin care and beauty products; the second day she uses no products; and the third day she uses “clean” products, which are allegedly free from phthalates and parabens.

When she receives her results, Nguyen is shocked by the high levels of phthalates in her urine on a “normal” day, when she uses all her regular products. She worries about her future fertility, given that has been using these products for a long time. The graph shows how the levels of toxins are much lower on the second day, and that level is maintained on the third day, when she uses clean cosmetics. Using such testing and self-experimentation to raise awareness of chemical toxicities is a good harm-reduction-from-below approach, not only because it fits with the way young people already tinker with chemicals and monitor effects in their bodies but also because it helps them overcome the limits of what they can directly feel and see.

Helping people “see” slow chemical toxicities may be informed by the bio-ethnographic approach being developed by Roberts and Sanz (2017). Their approach combines “two methodological bundles – ethnographic observation and biochemical sampling – in a synthetic analysis that understands environment interactions as always relational, contingent and constructed phenomena” (Roberts and Sanz 2017, p. 749). They point out that combining divergent methodologies and epistemologies is a slow process, but one that can bring together disparate practitioners and practices.

If we were to do a follow-up project to the ChemicalYouth project, we would seek to involve young people not only in the ethnography of their chemical consumption but also in generating understanding of the slow, less visible toxicities that make up their chemical regimes of living. Facing the daunting array of chemicals that make up our everyday lives, we would suggest that such studies are best done locally, as a way to put some spatial boundaries around the objects of inquiry.

Having provided ethnographic descriptions of young peoples’ situated chemical practices, and outlining the toxicities at stake, we now propose to expand our inquiry to include also less visible, unintentional environmental exposures. This might include examining the chemical safety of water, air, housing materials, working spaces, food, and soils that make up young people’s everyday lives. But we feel firmly that deciding what to examine should be co-determined by the young people who face the toxic risks, if only because this will also encourage them to act on the chemical regimes of their lives.

Toxicities involve complex feedback loops and emergent effects. Bio-ethnographic strategies can help us unravel some of the complexities, so as to prioritize societal actions that may reduce chemical harm. Focused bio-ethnographic studies can invite young people to measure the existence of toxins in the environments in which they live, observe seepage into and out of their bodies (as in the urine tests described above), while analyzing also the routes through which pollution takes place.

The ChemicalYouth project has developed collaborative ways of engaging young people in such research, to not only collect data but also write-up reports and publish scientific articles, and the distributed nature of this project has allowed for contrasting analyses across sites and collaborative analysis and action. A ChemicalYouth 2.0 could involve a wider range of researchers, advisors, and laboratories, in order to make more visible and tangible the multiple toxicities that make up young people’s everyday lives—taking our cue from the multiple intersecting strategies employed by activists and researchers in the field of endocrine disruption (Wylie 2018). ChemicalYouth 2.0 could thus take stock of the multitude of chemicals present in the commodities they consume, as well as the presence of toxins in their blood and urine, while surveying also the prevalence of benefits as well as adverse effects such as endocrine disruption, skin conditions, and respiratory problems.

We saw in Chapter 10.1007/978-3-030-57081-1_8 that similar collaborative research emerged around the testing of safety and efficacy of microdosing, which involved experimentation with a single category of chemicals. The challenge at stake is to broaden this collaboration to involve a wide range of chemicals, and to develop other ways of measuring both efficacy and potential toxicities. Roberts and Sanz (2017) point out that in practice bio-ethnography involves bridging diverse ways of knowing chemicals, while attending to “logistical minutiae and methodological challenges” (p. 751). We have experience in doing this and are ready to take on the next challenge. Collective advocacy, along with collaborative research to produce situated evidence of harm, can support efforts to implement the precautionary principles in policies to regulate chemicals, and to confront the regimes of invisibility that constrain effective regulatory action.

## Reducing Harm from Above

These harm-reduction-from-below efforts would gain much traction if they were reinforced by concerted efforts “from above” to unmask the invisibility forged by a range of societal actors. In pointing to the need for such a multi-pronged approach, we are inspired by the historical analysis conducted Gaudillière and Hess (2013), who describe the regulatory potentials of multiple stakeholders who co-produce regulatory regimes. We have highlighted the pragmatic approach, chosen by Amsterdam’s city government, to provide youth access to pill-testing facilities, and the work of the Trimbos Institute in developing youth-friendly educational materials. Such institutional efforts can help. A similar role is played by organizations like the Silent Spring Institute in testing urine, thereby giving people a valuable impetus for changing their everyday chemical regimes.

EU regulators too are taking action. In the introduction we pointed to the European Union’s Registration, Evaluation and Authorization of Chemicals (REACH) Policy, which has led to the banning of 1328 chemicals that are known or suspected to cause cancer, genetic mutations, reproductive harm, and/or birth defects from cosmetics. Finally, global treaties, such as the Framework Convention on Tobacco Control, can help hold nation states accountable for the implementation of policies and laws to prevent harm. Through this concerted action, misinformation on tobacco safety has been neutralized and tobacco consumption dramatically reduced across the globe.

Harm reduction from above strategies would also counter the harmful effects of white privileging that are reinforced by the making and marketing of skin-whitening products. These could include a ban on advertising skin creams and soaps, as such ads reinforce discriminatory policies that discipline young women into whitening their faces, as observed in the malls in Cagayan de Oro. Harm reduction from above would recognize the sexual health needs of youth, as well as the pragmatic ways they use emergency contraception to prevent unwanted pregnancies, achieve sexual hygiene through vaginal washes and penile tissues, and shape their (trans)gendered ways of being in the world. Harm reduction from above efforts could offer young people avenues for developing safer sexual health chemicals that meet their needs (Hardon et al. 2019).

Acute and slow toxicities can be reduced, and we have many tools: collective action by youth, harm reduction from below programs that build on young people’s self-regulation, collaborative research and development, responsible entrepreneurship, better science communication, multi-modal arts projects, transparent governance by food and drug authorities and consumer protection agencies with an aim of upholding the precautionary principle, and public scrutiny. These efforts begin by acknowledging the benefits that youth seek in their situated chemical practices.

## Accounting for Emergence

All harm reduction efforts, from below and above, need to also acknowledge the fluidity of chemical toxicity, which means examining the chains of events through which chemicals enter youths’ lives and accounting for always-emergent toxicities. In the case of Johnson’s Baby Powder, for example, it means accounting for the asbestos present in the mines where talc is extracted, and in the case of supplements it means attending to the regulatory space that allows manufacturers to make claims about a product’s metabolic benefits without being required to present evidence of its safety. In the case of e-cigarettes, it means attending to the reactions between lung cells and chemical vapors, examining nicotine content and the added flavors and thinners that may cause severe pathologies in the lungs of some users, which puts them at further risk for disease when a novel coronavirus emerges.

Attending to fluid toxicities also requires attention to corporate responses to societal trends and regulatory practices. We have seen across the chapters that when regulations are adopted by governments, societal actors respond (Black 2008). Users seek substitutes for their needs, while corporations can pay expensive lawyers and public relations firms to create smokescreens around the measurement of toxicity, in order to sow doubt about scientific evidence, and they frequently reinvent their products to circumvent regulation, just as Johnson and Johnson replaced talc with corn starch. When 180 states worldwide committed to stringent regulations on cigarettes sales and marketing, companies introduced e-cigarettes, referring to them as “ safer nicotine products,” as we saw in Chapter 10.1007/978-3-030-57081-1_3. They market e-cigarettes online through social influencers, making it harder for regulators to sanction.

Further attending to the fluidity of toxicity entails the monitoring of global market dynamics and global disparities in the “under-regulation of chemical hazards, and corporate impunity” (Lee 2020, p. 8). When people in France or the United States stop buying products associated with toxic risks, corporations seek new markets in the global South, as we saw in Chapter 10.1007/978-3-030-57081-1_3, where we described how the manufacturers of JUUL, faced with US sales restrictions, are now expanding the distribution of e-cigarettes in Indonesia, a densely populated country that has not endorsed the global treaty on tobacco control. Faced with diminishing sales due to toxicity concerns, Johnson and Johnson decided to discontinue sales of its Baby Powder in the United States. What will happen with the product in Indonesia and the Philippines, where the product is (still) very popular?

Attending to fluidity, finally, entails studying ways of knowing that generate new ways of understanding toxicities (Liboiron et al. 2018). Environmental researchers are calling for new ways of sensing chemicals, including long-term studies to measure the cumulative effects of toxins, which may lead to the reassessment of the safety and efficacy of chemicals in everyday life (Gabrys et al. 2016; Fortun et al. 2014; Shapiro 2015; Balayannis and Garnett 2020). The Center for the Health Assessment of Mothers and Children of Salinas (CHAMACOS) study, conducted by scholars at the University of California Berkeley, for example, measured the effects of organophosphates in children born to 600 pregnant Latino women in farming communities in Salinas Valley. The study determined that children’s cognitive development, as tested at age seven, decreased with higher amounts of pesticides used during their mothers’ pregnancies (Sanchez Barba 2020; Gunier et al. 2017). They found: “reduced IQ, attention deficit disorders, developmental delays, autism, and other pervasive developmental disorders correlated with exposures to organophosphates” (Sanchez Barba 2020, p. 4). Based on these studies, the U.S. Environmental Protection Agency concluded that there is no safe level of chlorpyrifos, one of the more commonly used organophosphates (Sanchez Barba 2020).

New ways of knowing chemicals may also lead to the re-evaluation of regulatory actions. In Chapter 10.1007/978-3-030-57081-1_8, we saw how LSD (a psychedelic drug that was banned in the 1960) is increasingly used by young people in very low dosages to enhance creativity at work. We described how this new microdosing practice was accompanied by collaborative studies to take stock of benefits and adverse effects, which involved users working together with academic researchers in online communities. These collaborations inspired new ways of doing placebo-controlled trials, in which users themselves were responsible for randomizing (Chapter 10.1007/978-3-030-57081-1_8). The evidence that is emerging from studies may result in new assessments of the risks of LSD, and new psychoactive products, which can then be appropriated by youth in their search for wellness, creativity, and productivity at work.

Harm reduction efforts need to be pragmatic and flexible in order to respond to the emergence of new knowledge, to innovations in ways of measuring toxicities and efficacies, to new products and revamped marketing strategies, and to revised regulatory assessments. And with the same dedication of purpose, they must simultaneously be alert to the creative ways that young people “do” chemicals in their ongoing quests for a good life.

## ChemicalYouth Ethnographers

**Fig. 9.6:**
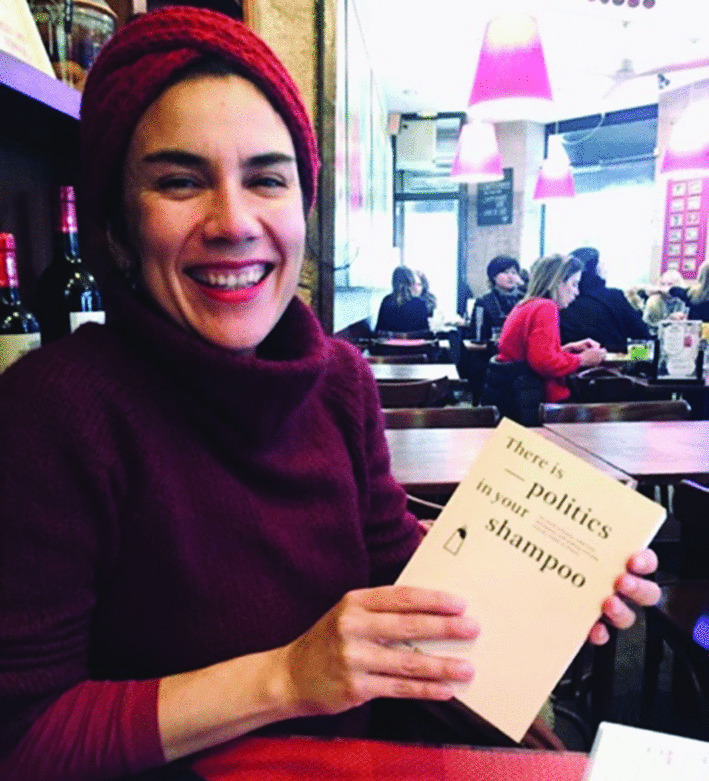
Mariana Rios Sandoval

Mariana Rios Sandoval’s PhD focused on the toxicity of everyday life and things in late industrial society, and on how young environmental activists in France made it visible, through political action and everyday practice, in order to fight it. This kind of toxicity was often articulated as the presence of endocrine-disrupting chemicals, but referred more generally to the myriad toxicants mediating the most ordinary, yet life-sustaining activities, such eating, keeping bodies and homes clean, having sex, and breathing. Mariana is now a Marie Curie post-doctoral fellow at CERMES3 with a project that explores how young French feminists integrate environmental considerations into their contraceptive practices (Fig. 9.6).

### Notes

1. Neuroscientists have recently argued that youths’ brains are wired to take risks (Bessant 2008), and social scientists have found that youth experience thrills when engaging in edgework (Lyng 1990). Our focused ethnographies suggest that young people’s chemical practices involve balancing benefits and harms. Our interlocutors had no appetite for living on the edge, and if they did so, it was usually caused by conditions of precarity beyond their control.
2. https://chemicalyouth.org/#/projects/mapping-chemicals-in-cagayan-de-oro.
3. https://vimeo.com/234326045.
4. https://vimeo.com/156004560 (password: fairandflawless) and https://vimeo.com/110724981 (password: brownisbeautiful).
